# Detection of flawed multiple-choice questions in preclinical medical education using item difficulty and discrimination indices: a six-year analysis

**DOI:** 10.1186/s12909-025-08204-5

**Published:** 2025-12-01

**Authors:** Varanya Srisomsak, Chantacha Sitticharoon, Issarawan Keadkraichaiwat, Sunan Meethes, Inpreeya Inpaen

**Affiliations:** 1 Department of Physiology, Faculty of Medicine Siriraj Hospital, Mahidol University, 2 Wanglang Rd., Siriraj, Bangkok Noi, Bangkok, Thailand 10700; 2 Education department, Faculty of Medicine Siriraj Hospital, Mahidol University, 2 Wanglang Rd., Siriraj, Bangkok Noi, Bangkok, Thailand 10700

**Keywords:** Exam, Analysis, Point biserial coefficient, Item difficulty, Exam threshold

## Abstract

**Background:**

MCQ exams may include flawed items affecting validity. Psychometric indicators such as item difficulty (*p*-value) and point-biserial coefficient (r_pb_-value) are widely used to identify problematic questions. Evidence on using *p*-value (< 0.25) and/or r_pb_-value thresholds (< 0) to detect flawed items remains limited. This study aimed to provide a proof-of-concept using a large, real-world dataset, evaluating how often flawed items were missed when relying solely on static thresholds.

**Methods:**

Exam analyses from 32 preclinical courses (academic years 2017–2022) were reviewed. Items meeting predefined thresholds were flagged, while all items were manually reviewed when the most frequently chosen answer was not the keyed correct answer or when multiple options had similar *p*-values. Flagged items were sent to course directors for verification, and only confirmed items were recorded as corrections.

**Results:**

Among 236 exams, 59 (25.0%) required corrections, with at least 1 corrected item. Of 14,238 total items, 81 (0.6%) required correction, most due to ‘multiple-answers’ (46.9%), followed by ‘wrong-answer’ (40.7%), awarding points to all choices (‘all-choices’) (7.4%), and ‘item-removal’ (4.9%) causes. Of 77 corrected items with available *p*- and r_pb_-values (excluding ‘item-removal’), 66 (85.7%) met thresholds, while 11 (14.3%) had *p*-value ≥ 0.25 and r_pb_-value ≥ 0, indicating thresholds missed 14.3%. Corrected items had significantly lower *p*- and r_pb_-values than uncorrected items (*P* < 0.001). Item correction status showed negative correlations with both *p*- and r_pb_-values, suggesting flawed items tended to be more difficult and less discriminative. For the ‘wrong-answer’ and ‘multiple-answers’ causes, the actual correct answers had higher *p*-values than the initially designated ones (*P* < 0.001). For the ‘wrong-answer’ cause, the actual correct answers had higher r_pb_-values (*P* < 0.001), while they were comparable in the ‘multiple-answers’ cause, highlighting flaws even with unremarkable r_pb_-values.

**Conclusion:**

While *p*- and/or r_pb_-value thresholds detected 85.7% of flawed items, 14.3% were missed, underscoring that static thresholds are insufficient to ensure item quality. This study provides empirical evidence supporting the integration of quantitative indices with expert review in exam evaluation. Incorporating items where the keyed answer has a *p*-value less than or similar to distractors may improve detection but increase workload, reflecting a trade-off between accuracy and feasibility.

## Background

Multiple-choice questions (MCQs) are one of the most commonly employed testing formats for assessment because they enhance reliability, validity, and ease of scoring [1]. Furthermore, MCQs have become the predominant method for assessing knowledge and proficiency of medical students [2]. They serve as an effective tool for assessing students’ knowledge, higher cognitive skills, application, and analytical abilities [1]. Assessment is an important component of teaching and learning, with particular importance in continued monitoring of learning activities and in providing feedback to students and teachers [2].

Following the administration of an MCQ exam, the quality of each item and the overall exam can be evaluated and improved. When using questions and exams as assessment benchmarks, failure to standardize them may result in students receiving higher or lower marks than they truly deserve, undermining the learning process in several ways [3]. This not only diminishes society’s trust in the educational system but also risks producing underqualified doctors. Implementing standardized tests through a process known as test and item analysis could mitigate this issue. Test and item analysis involves two main tasks, evaluating individual questions and reviewing the entire test [3].

Item analysis involves gathering, summarizing, and utilizing data from students’ responses to evaluate the quality of MCQs. Conducting item analysis is key to ensuring the quality of MCQs presented to students, relying on the responses provided by the examinees. Questions that are excessively difficult or overly simplistic may require revision or elimination. Determining whether to revise questions is based on performance characteristics including item difficulty (also referred to as the *p*-value), discrimination index (DI), point biserial coefficient (r_pb_-value), and effectiveness of distractors [4–6]. A well-constructed test should incorporate essential psychometric qualities by balancing proportions of easy, moderate, and difficult items to enhance validity and ensure that assessments capture a range of student abilities [7].

The item difficulty represents the proportion of examinees who correctly answer a specific test item, with values ranging from 0 to 1, where the higher item difficulty indicates an easier question [6]. A *p*-value < 0.3 indicates a too difficult question, 0.3–0.7 suggests good/acceptable/average difficulty, and > 0.7 reflects a too easy item, while an item difficulty 0.5–0.6 is considered excellent/ideal [8]. When the average item difficulty across all items in an exam falls below 0.3, it may indicate that the exam as a whole is very difficult [8].

The r_pb_-value, ranging from − 1 to + 1, is an indicator of an item’s ability to distinguish between higher- and lower-performing students, representing the relationship between item performance and overall test performance [4]. When r_pb_-values are averaged across items, the resulting value can represent the overall discriminative quality of the exam [4]. Tests with a positive r_pb_-value indicate that individuals with higher overall test performance tend to answer the item correctly more often than individuals with lower overall test performance, demonstrating effective discrimination [4, 5]. Conversely, if the test has a negative r_pb_-value, it indicates that individuals with higher overall test performance tend to answer the item incorrectly more often than those with lower overall test performance, thereby failing to discriminate effectively [4, 5].

The r_pb_-value below 0 indicates flawed items, 0–<0.15 is considered poor, 0.15–<0.25 is recommended, and ≥ 0.25 is good, reflecting increasing levels of item discrimination [4]. If the item has a negative r_pb_-value, it should be discarded, as it may indicate a problem in teaching and learning that causes students to misunderstand or fail to comprehend the subject being taught [9].

Effectiveness of distractors serves as a tool to evaluate whether an item is well constructed. Any distractor chosen by fewer than 5% of students is classified as a non-functioning distractor (NF-D). Ideally, students with lower overall scores, indicating less mastery of the subject, should select distractors more frequently, while high-scoring students should reject them more often in favor of the correct option. Analyzing distractors facilitates the identification of errors, allowing for their revision, replacement, or removal [10].

After each MCQ exam of the preclinical courses at the Faculty of Medicine Siriraj Hospital, the Education Department conducts exam analysis with set thresholds, which are a *p*-value < 0.25 and/or an r_pb_-value < 0, to identify potential flawed items that require corrections. However, after thorough observation, some item corrections were observed even when the analysis did not meet these thresholds. Additionally, the correct answer was not most often selected by students, and there were instances of multiple correct choices, with the *p*-value of the first correct choice being less than or close to the others’ choices. Relying solely on these thresholds may result in the under-detection of flawed items, as some flawed items may not affect difficulty or discrimination indices and thus may escape detection [11]. The Education Department has brought attention to these issues, leading to requests for further verification from the course director when the *p*-value of the correct choice is lower or similar to others. Confirmations from the course director revealed instances where the initially designated correct answer was incorrect, necessitating changes, or where questions had multiple correct answers, or none of the provided options were correct. Frequently, the designated corrected answers did not initially align with the predefined *p*-value and r_pb_-value thresholds but matched the Education Department’s additional observations. Thus, using only threshold criteria may lead to the under-detection of flawed items.

In previous studies, only the ranges of *p*-value and r_pb_-value associated with item quality were presented. However, research has been limited in systematically investigating the causes of item corrections in relation to their psychometric properties, particularly how *p*-value and r_pb_-value correspond to each specific cause, and in evaluating the effectiveness of *p*-value and r_pb_-value for detecting flawed items. Accordingly, our research questions were conceptually framed to address two issues informed by measurement theory: (1) how item corrections relate to item difficulty and discrimination; and (2) whether conventional thresholds (< 0.25 for *p*-value, < 0 for r_pb_-value) sufficiently capture flawed items or may miss certain problematic cases. This study therefore aimed to provide empirical evidence from a large, real-world exam dataset from preclinical courses between academic years 2017–2022 to: (1) identify the causes of item corrections and their associated *p*-value and r_pb_-value, both with and without corrections; and (2) determine the effectiveness of *p*-value and r_pb_-value thresholds in detecting flawed items. By addressing these aims, our study sought to provide a proof-of-concept regarding how often flawed items may be missed when relying solely on statistical thresholds, thereby supporting the development of future guidelines to improve flawed MCQ detection and enhance exam quality in medical education, while balancing detection accuracy with reviewer workload.

## Materials and methods

### Curriculum, study protocol, and data collection

The Doctor of Medicine Program at the Faculty of Medicine Siriraj Hospital, Mahidol University, has a duration of 6 years, divided into preclinical years (medical years 1–3) and clinical years (medical years 4–6).

Exam analysis was conducted for preclinical courses, covering the academic years 2017–2022. At our institution, the academic years 2017–2022 were organized as follows: medical year 1 from August to May; medical year 2 from August to May or June in 2017–2021, and from June to March in 2022; and medical year 3 from June to April. All exams were administered online using the SELECx-Exam, a Moodle-based platform integrated with the Safe Exam Browser, which locks the screen and prevents students from accessing other applications during the examination.

Almost all MCQ items consisted of 1 correct option and 4 distractors, although a very small number of items contained fewer distractors due to limitations in item construction. The allotted time was set as an average of 1.5 min/item, applied to the entire exam rather than to individual items. If a student did not complete all questions within the time limit, the system automatically saved the responses up to that point, and the score was calculated based on completed items only. Additional time was not granted, except in cases of computer or software malfunction. The exam timer began once the student logged into the system.

The exam results were used for summative decisions. From academic years 2017–2019, exams were graded on an A-F scale. During the Coronavirus Disease 2019 (COVID-19) period (academic years 2020–2021), grading varied by course, with either the A-F scale or pass/fail depending on whether reliable discrimination was feasible. In academic years 2021–2022, medical year 1 and year 2 courses adopted a pass/fail system for all subjects, with the option of awarding an ‘Outstanding’ (O) grade when students achieved ≥ 80% and when the O grade could be determined with confidence. A score of ≥ 60% was considered passing, awarding a ‘Satisfactory’ (S) grade. Students who did not pass on the first attempt received a ‘Not Yet Pass’ (X) grade and were given one remediation opportunity before a final pass/fail decision. Importantly, the presence of flawed items could compromise the fairness of these grading decisions, whether A-F, Outstanding, or pass/fail.

### Exam analysis

Examinations of all preclinical courses were sent to the Education Department for centralized analysis. The MCQ exams were analyzed using an exam analysis program developed in-house by the department’s programmer. The analysis includes metrics at the exam, item, and choice levels. At the exam level, the parameters analyzed include the number of items, number of students, mean score (calculated as the average of student scores for each exam), standard deviation (SD), mode, maximum score, minimum score, reliability coefficient, and standard error of measurement (SEM). At the item (question) level, *p*- and r_pb_-values are calculated for each item based on the initially designated correct answer, representing the difficulty and discrimination of the item. At the choice (option) level, *p*- and r_pb_-values are calculated separately for each option, including both the keyed correct answer and distractors, to examine response patterns and identify potential issues such as distractor attractiveness or miskeying. The item-level *p*- and r_pb_-values are then summarized at the exam level as average, maximum, and minimum values.

The item difficulty is calculated by the following formula [12]:

$${p}-\mathrm{value=}\frac{\mathrm{number}\;\mathrm{of}\;\mathrm{students}\;\mathrm{answer}\;\mathrm{correctly}}{\mathrm{total}\;\mathrm{number}\;\mathrm{of}\;\mathrm{students}\;\mathrm{answer}\;\mathrm{that}\;\mathrm{item}}$$

The r_pb_-value is determined using the formula [9, 12]:

$${\mathrm r}_{\mathrm{pb}}-\mathrm{value}=\frac{{\mathrm M}_{\mathrm p}-{\mathrm M}_{\mathrm q}}{\mathrm{SD}}\;\sqrt{\mathrm{pq}}$$

M_p_ = The average total score of test takers who answer the question correctly.

M_q_ = The average total score of test takers who answer the question incorrectly.

SD = Standard deviation of test scores.

p = Proportion of test takers who answer the question correctly out of all test takers.

q = Proportion of test takers who answer the question incorrectly out of all test takers.

For each MCQ exam, every item is reviewed by the education staff based on standard indicators. Items with a *p*-value < 0.25 and/or r_pb_-value < 0 are automatically flagged for potential correction. In addition, all items are manually reviewed regardless of threshold values, with particular attention to cases where the correct answer is not the most frequently selected option or where multiple choices show similar *p*-values. This comprehensive review ensures that errors not detected by psychometric thresholds can still be identified. Flagged items are then forwarded to course directors for verification. Item corrections are officially recorded only after confirmation by the course director, while exam corrections refer to any exam that includes at least 1 item requiring correction. All item statistics reported in the Results and Discussion sections refer to values calculated from the initially designated correct answer (i.e., pre-correction).

In the most recent academic year (2022), all items with their corresponding *p*- and r_pb_-values were recorded and analyzed to characterize items with and without corrections. Data from other academic years were not included due to the extensive size of the database. Furthermore, the correlations between item correction status and their associated *p*- and r_pb_-values were examined to determine the relationship between item correction status and item-level psychometric properties.

### Statistics

The Statistical Package for the Social Sciences (SPSS) version 18 was used for statistical analysis. Descriptive statistics, including frequency, mean, range, and percentage of *p*- and r_pb_-values, were calculated, with subgroup analyses based on academic year, medical year, and the causes of item corrections. Item corrections were categorized into 4 groups according to *p*- and r_pb_-value thresholds: (1) *p*-value < 0.25 and r_pb_-value < 0, (2) *p*-value < 0.25 and r_pb_-value ≥ 0, (3) *p*-value ≥ 0.25 and r_pb_-value < 0, and (4) *p*-value ≥ 0.25 and r_pb_-value ≥ 0. This categorization was used to examine the distribution of items across these threshold groups and to evaluate the effectiveness of applying each threshold individually or in combination to identify flawed items. Differences in the proportions of corrected items between causes were evaluated using the chi-square test. Additionally, comparisons of *p*- and r_pb_-values between the initially designated correct choice and the actual correct choice(s) were conducted, based on pre-correction values of the initially designated key, using a paired-sample t-test, while comparisons between items with and without corrections were performed using a non-parametric test. Correlation analysis between exam or item corrections and *p*- and r_pb_-values was performed using Pearson’s product-moment correlation coefficient for continuous data and Spearman’s rank correlation coefficient for ranked data.

## Results

### Descriptive analysis of total exams, exams without corrections, and exams with corrections

Descriptive analysis of total exams, exams without corrections, and exams with corrections is shown in Table 1.

**Table 1:**
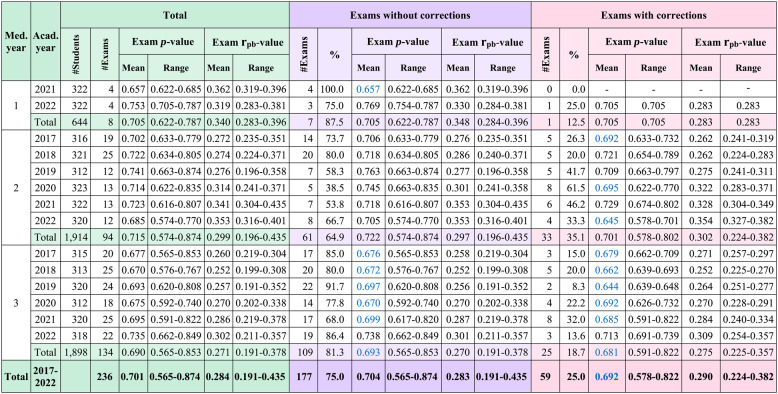
Descriptive analysis of total exams, exams without corrections, and exams with corrections

* Med. year* Medical year, *Acad. year *Academic year, *# * Number, Blue text represents the mean p-value within the acceptable range

In medical year 1, exams without corrections were in the mean acceptable *p*-value range in 1 academic year and in the easy *p*-value range in the other, while the only exam with corrections was in the easy *p*-value range (Table 1). In medical year 2, exams without corrections were consistently in the easy *p*-value range across all 6 years, whereas exams with corrections were divided equally between the easy and acceptable *p*-value ranges (Table 1). In medical year 3, both corrected and uncorrected exams were in the acceptable *p*-value range in 5 years (83.3%) and in the easy *p*-value range in 1 year (16.7%) (Table 1). Overall, exams without corrections more frequently fell within the easy *p*-value range compared with exams with corrections (Table 1). Across all medical years, the mean r_pb_-values for total, corrected, and uncorrected exams were consistently within the good range of item discrimination (Table 1).

### Distribution of exam correction status categorized by *p*- and/or r_pb_-value ranges

**Fig. 1:**
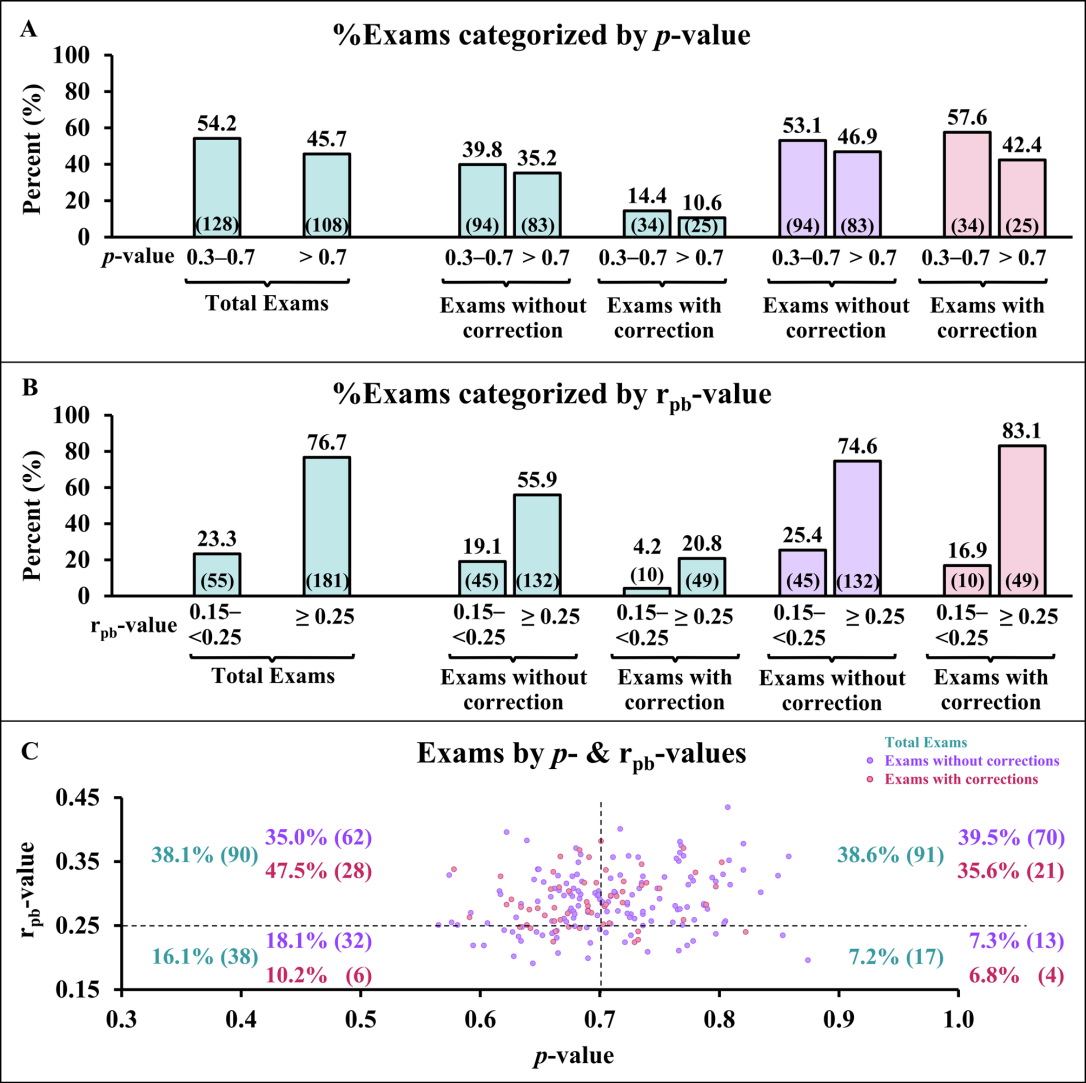
Distribution of exam correction status categorized by *p*- and/or r_pb_-value ranges. Fig. 1**A**: Percentage of exams categorized by *p*-value ranges; Fig. 1**B**: percentage of exams categorized by r_pb_-value ranges. For Fig. 1**A** and **B**, green bars represent the percentage of total exams, purple bars represent the percentage of exams without corrections, and pink bars represent the percentage of exams with corrections. Fig. 1**C**: scatter plots and percentage of exams by *p*- and r_pb_-values. Green text represents the total number of exams, purple plots/text represent exams without corrections, and pink plots/text represent exams with corrections with percentages calculated based on each correction category. Numbers in parentheses represent the number of exams in each category shown in the bars.

The distribution of exam correction status categorized by *p*- and/or r_pb_-value ranges is shown in Fig. 1.

Across all exams, the distribution of *p*-values showed that exams with and without corrections were similarly distributed between the acceptable (0.3–0.7) and very easy (> 0.7) ranges, with no clear separation between 2 groups (Fig. 1A). For r_pb_-values, most exams fell within the good discrimination range (≥ 0.25), again with comparable proportions of corrected and uncorrected exams (Fig. 1B). When *p*- and r_pb_-values were combined, corrected and uncorrected exams were present across all categories with overlapping distributions (Fig. 1C). Overall, these results indicate that exam-level averages of *p*- and r_pb_-values did not distinguish exams requiring correction from those without corrections.

### Distribution of corrected items categorized by causes

The distribution of corrected items categorized by causes is shown in Table 2.

**Table 2:**
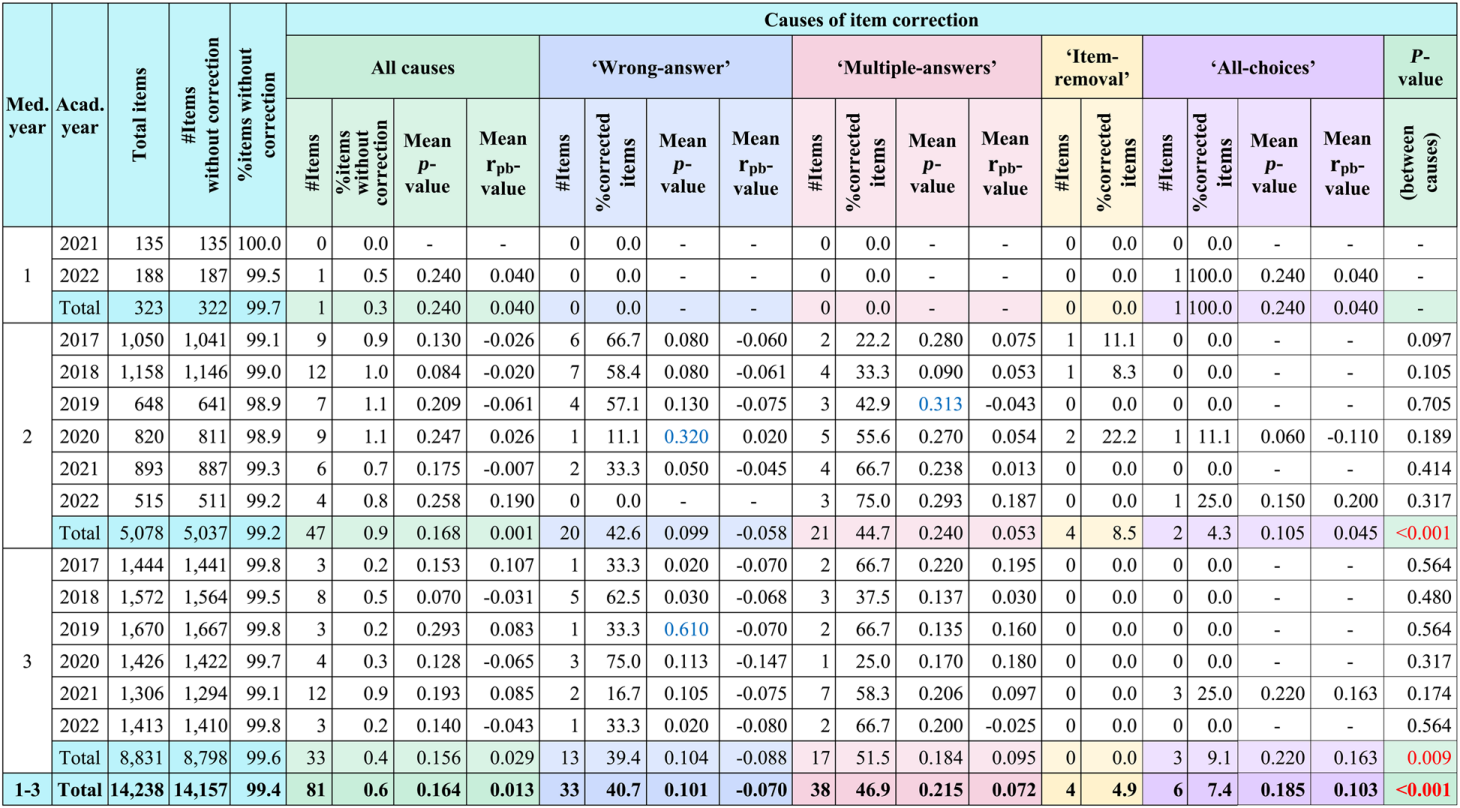
Distribution of corrected items categorized by causes

*Med. year* Medical year, *Acad. year *Academic year, *# * Number, ‘All-choices’ awarding points to all choices, Blue text represents the mean *p*-value within the acceptable range, Red text represents a statistically significant difference

The proportions of corrected items differed significantly among causes of correction in medical year 2 (χ², *P* < 0.001), medical year 3 (χ², *P* = 0.009), and overall across medical years (χ², *P* < 0.001) (Table 2). Despite the small number of corrected items, the statistical test still revealed significant variation in the distribution pattern of causes in medical year 2, medical year 3, and overall across medical years. Across all medical years, the mean *p*-value of corrected items, regardless of cause, fell within the very difficult range (Table 2). Subgroup analysis showed exceptions, with the acceptable *p*-value range observed for the ‘wrong-answer’ cause in medical year 2 during academic year 2020 and for the ‘multiple-answers’ cause in medical year 2 during academic year 2019, while in medical year 3 during academic year 2019 the ‘wrong-answer’ cause also fell within the acceptable range (Table 2). Mean r_pb_-values for corrected items included both positive and negative values across all causes (Table 2). For the ‘wrong-answer’ cause, negative r_pb_-values were more common, occurring in 80% of cases in medical year 2 and in all cases in medical year 3 (Table 2). In contrast, the ‘multiple-answers’ cause more often showed positive r_pb_-values, occurring in 83% of cases in both medical year 2 and medical year 3 (Table 2).

### Descriptive analysis of total items, items without corrections, and items with corrections in academic year 2022

Descriptive analysis of total items, items without corrections, and items with corrections in academic year 2022 is shown in Table 3.

**Table 3:**
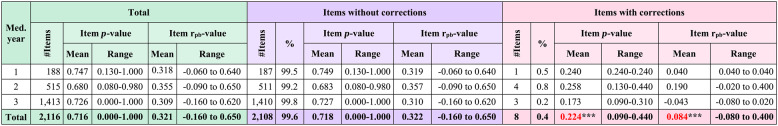
Descriptive analysis of total items, items without corrections, and items with corrections in academic year 2022

*Med. year* Medical year, *#* Number, Red text represents a statistically significant difference, ***P < 0.001 compared to items without corrections

In academic year 2022, corrections occurred in only a very small proportion of items, ranging from 0.2 to 0.8% across medical years (Table 3). In every medical year, corrected items had lower mean pre-correction *p*- and r_pb_-values than uncorrected items (Table 3). When combined across all medical years, corrected items accounted for only 0.4% of the total, and their mean pre-correction *p*- and r_pb_-values were significantly lower (*P* < 0.001), indicating that flawed items prior to correction tended to be more difficult and less discriminative than items that did not need correction (Table 3).

### Correlations between exam/item corrections and *p*- and r_pb_-values

Correlations between exam/item corrections and *p*- and r_pb_-values are shown in Table 4.

**Table 4:**
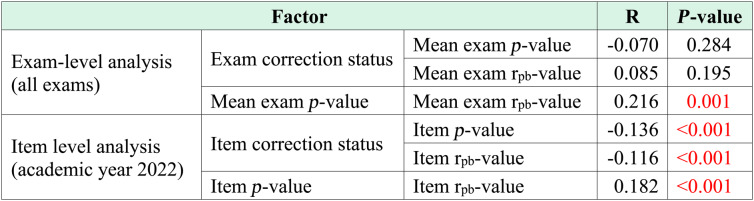
Correlations between exam/item corrections and *p*- and r_pb_-values

*R* correlation coefficient, Red text represents a statistically significant difference

At the exam level, correction status showed no significant correlation with either mean *p*-values or mean r_pb_-values (Table 4). A positive correlation was observed between mean exam *p*- and r_pb_-values, although the strength of the association was low (Table 4). At the item level in academic year 2022, correction status was negatively correlated with both *p*- and r_pb_-values, while a positive correlation was found between item *p*- and r_pb_-values (Table 4).

### Distribution of corrected items categorized by *p*- and/or *r*_pb_-value ranges

**Fig. 2:**
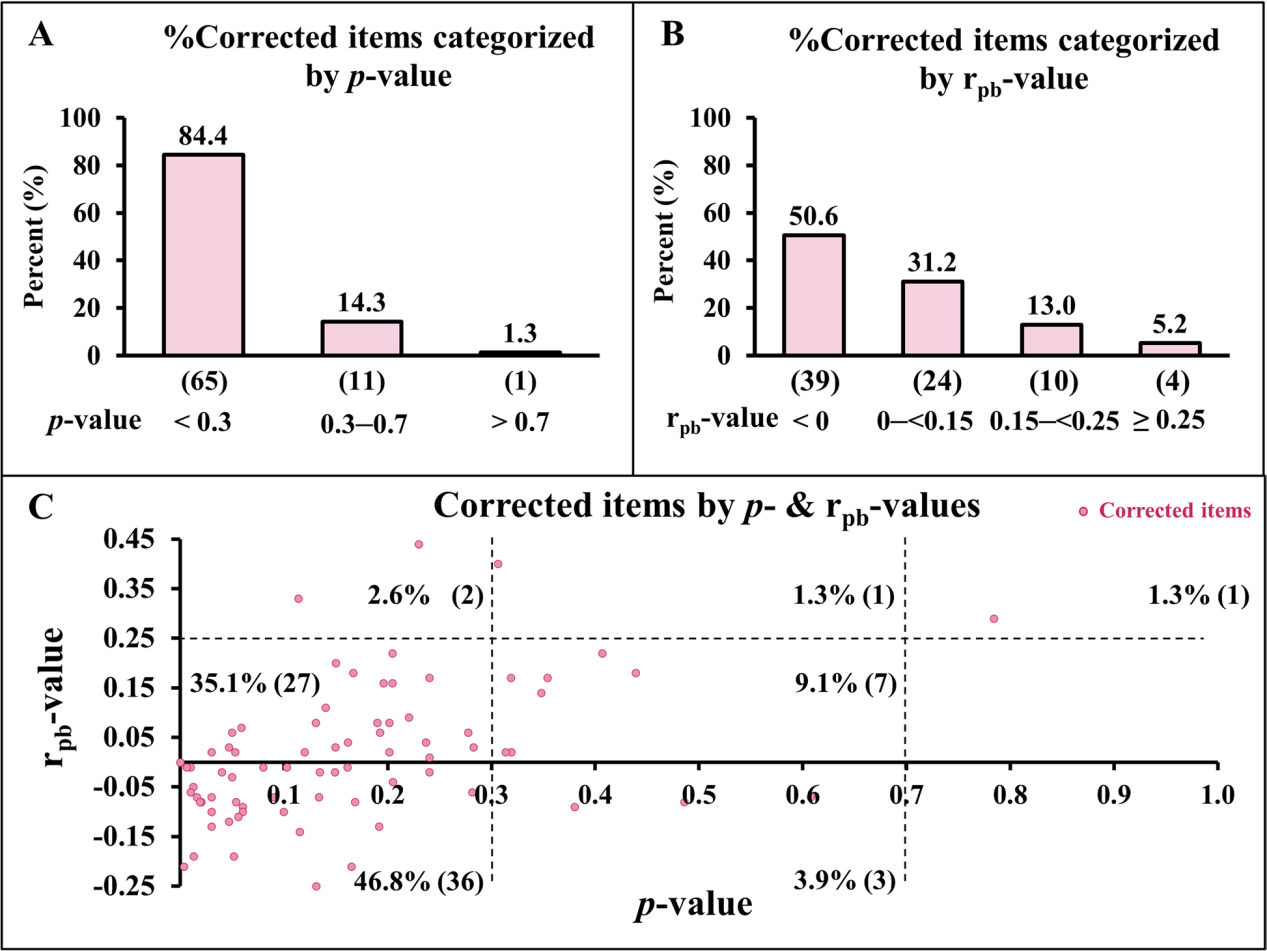
Distribution of corrected items categorized by *p*- and/or r_pb_-value ranges. Fig. 2**A**: Percentage of corrected items categorized by *p*-value ranges; Fig. 2**B**: percentage of corrected items categorized by r_pb_-value ranges. For Fig. 2**A** and **B**, numbers in parentheses indicate the number of items. Fig. 2**C**: scatter plots showing corrected items by *p*- and r_pb_-values.

The distribution of corrected items categorized by *p*- and/or r_pb_-value ranges is shown in Fig. 2.

Figure 2 applied a *p*-value cutoff of 0.30 to provide broader categorical ranges for visualization. Among the 77 corrected items, the majority (84.4%) were in the very difficult range with a *p*-value < 0.3, while only 14.3% fell within the acceptable range (0.3–0.7) and 1.3% were in the easy range (> 0.7) (Fig. 2A). Regarding discrimination, half of the corrected items (50.6%) showed negative r_pb_-values, and nearly one-third (31.2%) had poor discrimination (< 0.15). Only a small proportion (5.2%) demonstrated good discrimination (≥ 0.25) (Fig. 2B). When difficulty and discrimination indices were considered together, nearly half (46.8%) of the corrected items combined a very low *p*-value with a negative r_pb_-value, and over one-third (35.1%) had very low *p*-values with marginal r_pb_-values (Fig. 2C). Overall, flawed items were predominantly very difficult and poorly discriminative.

### Distribution of corrected items categorized by *p*- and r_pb_-value thresholds

The distribution of corrected items categorized by *p*- and r_pb_-value thresholds is shown in Table 5.

**Table 5:**
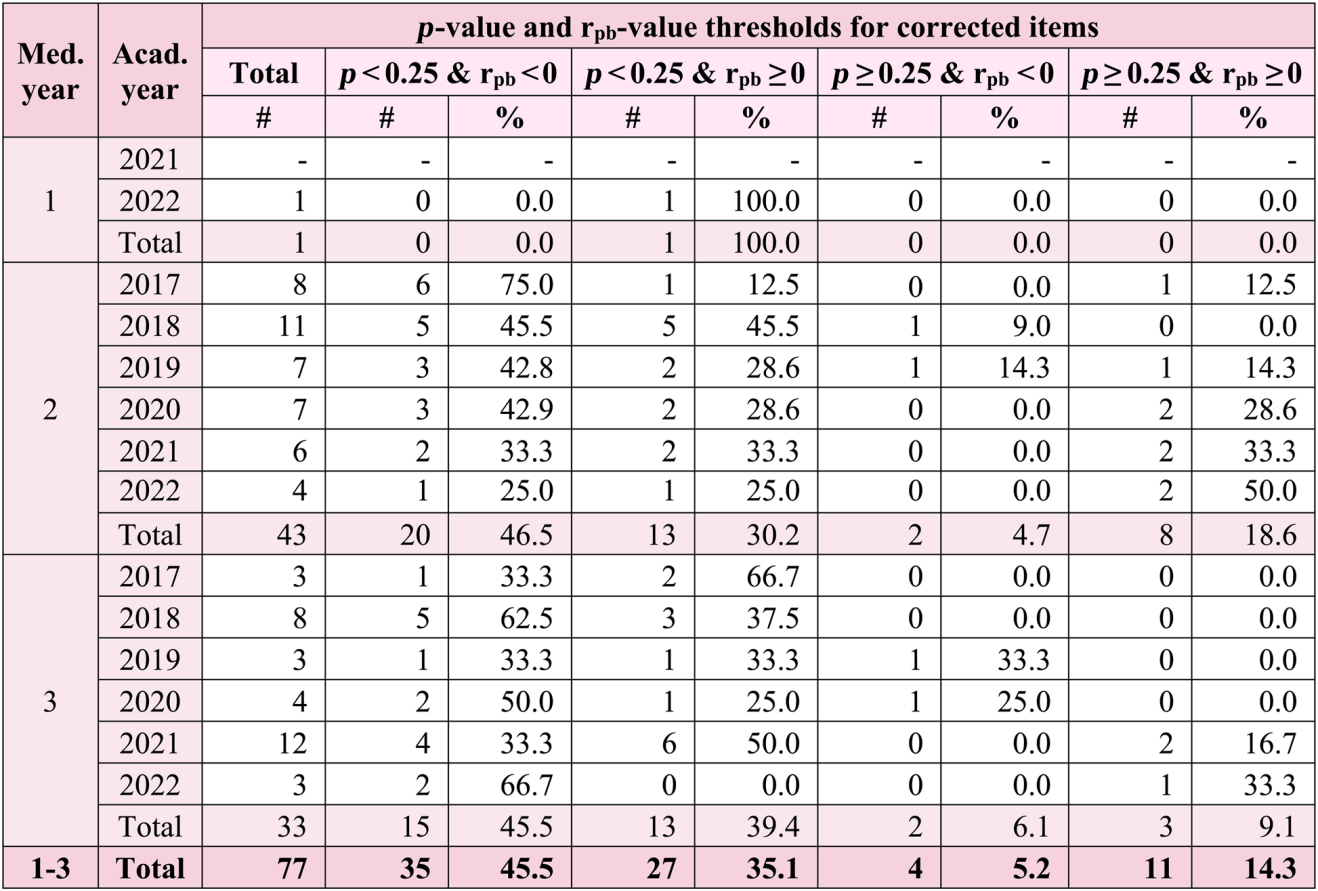
Distribution of corrected items categorized by *p*- and r_pb_-value thresholds

* Med. year * Medical year, *Acad. year* Academic year, *#* Number

The distribution of corrected items categorized by *p*- and r_pb_-value thresholds, using the predefined *p*-value cutoff of 0.25 specified in our analysis protocol to flag potentially flawed items, is shown in Table 5. In medical year 1, the only corrected item was classified as having a *p*-value < 0.25 with an r_pb_-value ≥ 0 (Table 5). In medical years 2 and 3, as well as overall, the largest proportion of corrected items was in the *p*-value < 0.25 with r_pb_-value < 0 range (45.5%), followed by *p*-value < 0.25 with r_pb_-value ≥ 0 (35.1%) (Table 5). Smaller proportions were observed in *p*-value ≥ 0.25 with r_pb_-value ≥ 0 (14.3%) and the smallest proportion in *p*-value ≥ 0.25 with r_pb_-value < 0 (5.2%) (Table 5).

### Distribution of corrected items categorized by causes of correction and *p*- and r_pb_-value thresholds

**Fig. 3:**
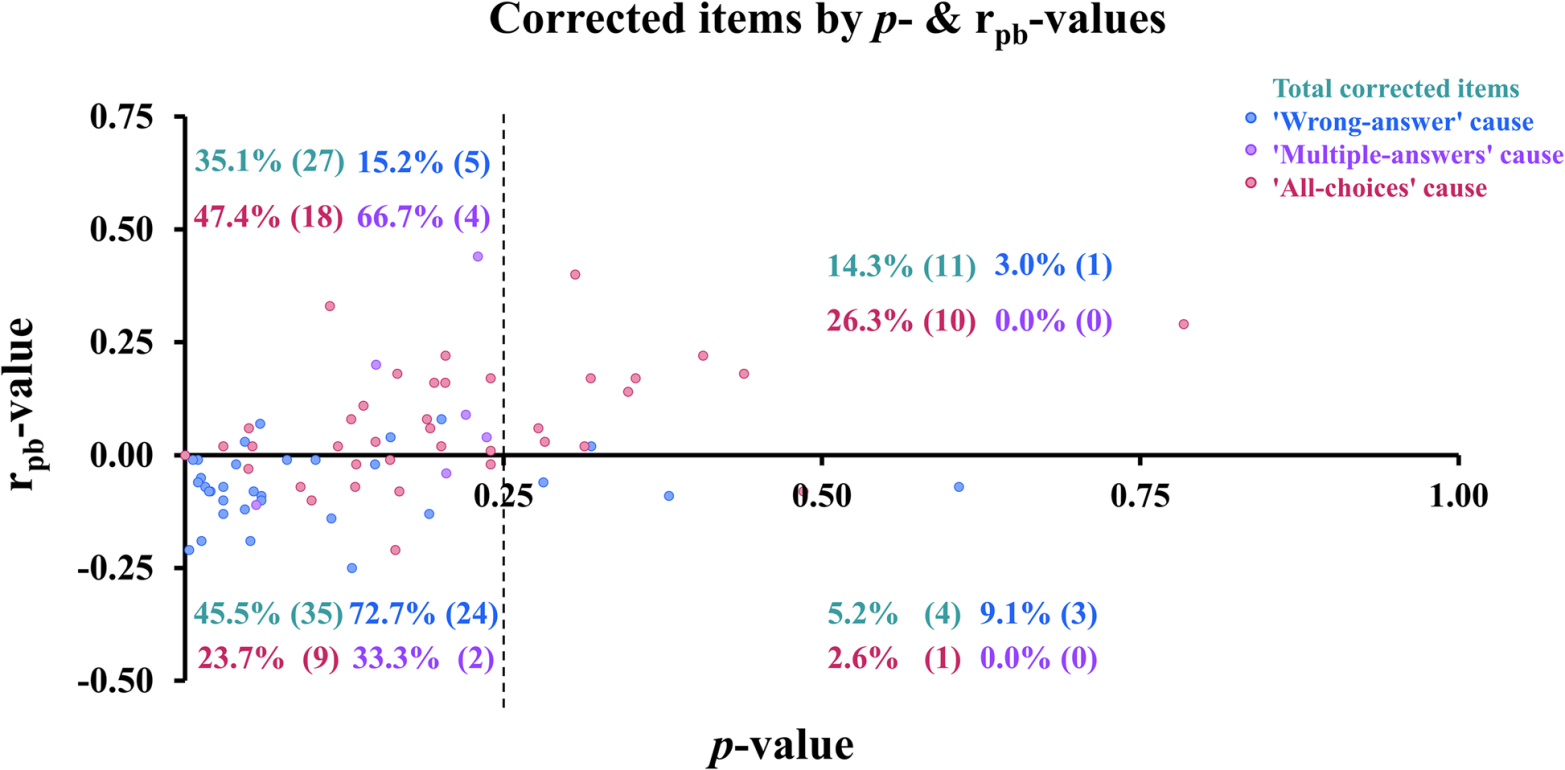
Distribution of corrected items categorized by causes of correction and *p*- and r_pb_-value thresholds. Fig. 3 Scatter plots showing corrected items by *p*- and r_pb_-value thresholds. Numbers in parentheses indicate the number of items. Green text represents the percentage of total corrected items, blue text represents the percentage of corrected items with the ‘wrong-answer’ cause, pink text represents the percentage of corrected items with the ‘multiple-answers’ cause, and purple text represents the percentage of corrected items with the awarding points to all choices (‘all-choices’) cause.

The distribution of corrected items categorized by causes of correction and *p*- and r_pb_-value thresholds is shown in Fig. 3.

Figure 3 applied a *p*-value cutoff of 0.25, consistent with the analysis protocol, to categorize corrected items by cause and by *p*- and r_pb_-value thresholds. Overall, nearly half of corrected items (45.5%) were classified as *p*-value < 0.25 with r_pb_-value < 0, followed by 35.1% in *p*-value < 0.25 with r_pb_-value ≥ 0, while fewer items fell into the higher *p*-value categories (14.3% in *p*-value ≥ 0.25 with r_pb_-value ≥ 0 and 5.2% in *p*-value ≥ 0.25 with r_pb_-value < 0) (Fig. 3). When analyzed by cause, the majority of ‘wrong-answer’ items (72.7%) clustered in the *p*-value < 0.25 with r_pb_-value < 0 range, whereas ‘multiple-answers’ items were more often distributed in *p*-value < 0.25 with r_pb_-value ≥ 0 (47.4%) or *p*-value ≥ 0.25 with r_pb_-value ≥ 0 (26.3%) (Fig. 3). Items corrected by awarding points to all choices (‘all-choices’) were fewer, but most (66.7%) were found in *p*-value < 0.25 with r_pb_-value ≥ 0 (Fig. 3). Overall, corrected items generally showed low *p*-values, with r_pb_-values tending to be negative for ‘wrong-answer’ but positive for ‘multiple-answers’ and ‘all-choices’ (Fig. 3).

### Comparisons of *p*- and r_pb_-values for corrected items between the initially designated correct answer and the actual or additional correct answers

**Fig. 4:**
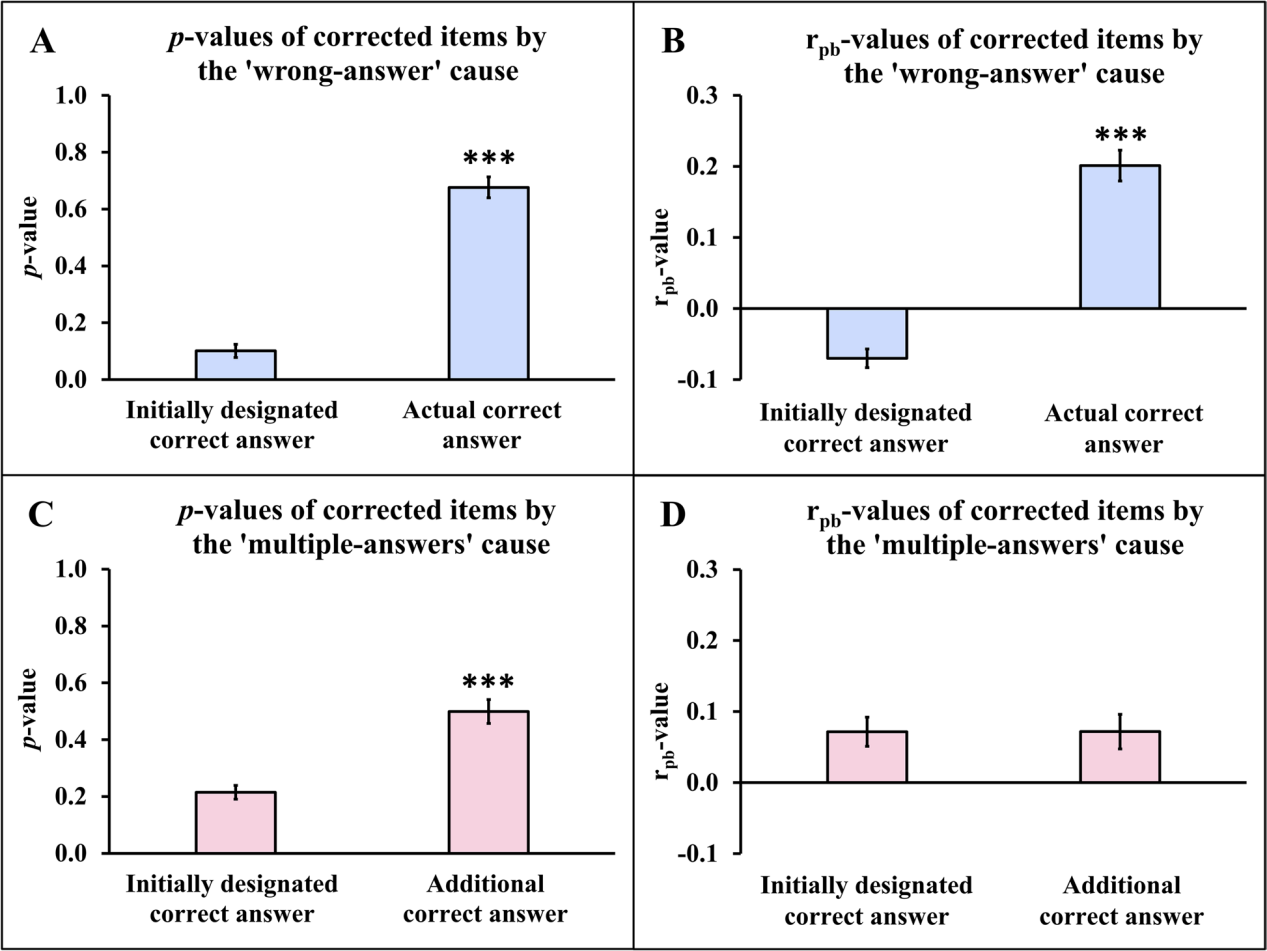
Comparisons of *p*- and r_pb_-values for corrected items between the initially designated correct answer and the actual or additional correct answers. Fig. 4**A**: Comparison of *p*-values for corrected items between the initially designated correct answer and the actual correct answer for the ‘wrong-answer’ cause; Fig. 4**B**: comparison of r_pb_-values for corrected items between the initially designated correct answer and the actual correct answer for the ‘wrong-answer’ cause; Fig. 4**C**: comparison of *p*-values for corrected items between the initially designated correct answer and the additional correct answer for the ‘multiple-answers’ cause; and Fig. 4**D**: comparison of r_pb_-values for corrected items between the initially designated correct answer and the additional correct answer for the ‘multiple-answers’ cause.

Comparisons of *p*- and r_pb_-values for corrected items between the initially designated correct answer and the actual or additional correct answers are shown in Fig. 4.

For the ‘wrong-answer’ cause, the actual correct answer exhibited a significantly higher pre-correction *p*-value (Fig. 4A) and r_pb_-value (Fig. 4B) compared to the initially designated correct answer (*P* < 0.001 both). Additionally, for the ‘multiple-answers’ cause, the pre-correction *p*-value of the additional correct answer(s) was significantly higher than that of the initially designated correct answer (*P* < 0.001) (Fig. 4C), while no significant difference in pre-correction r_pb_-values was observed between these answers (Fig. 4D).

## Discussion

This study was designed as a proof-of-concept, drawing on empirical evidence from a large, real-world exam dataset of preclinical courses spanning 6 academic years (2017–2022). To our knowledge, research has been limited in systematically examining the causes of MCQ item corrections in relation to their psychometric properties and in assessing the correspondence of *p*- and r_pb_-values to specific causes. The study also evaluated the role of statistical thresholds in detecting flawed items, with particular attention to their effectiveness.

At the exam level, most tests demonstrated an acceptable difficulty range, slightly above 50%, with a lower proportion categorized as very easy (Fig. 1A). This overall pattern aligns with previous reports in medical students, where exam-level indices typically indicated acceptable quality [13–15]. For r_pb_-values (Fig. 1B), ~ 75% of exams fell within the good range, while ~ 25% were in the recommended range. Similar findings have been reported in pharmacy students, suggesting that most exams, when considered as a whole, demonstrate good point-biserial performance [4].

Although both exam- and item-level data were analyzed and reported, exam-level data were presented mainly to contextualize aggregate patterns rather than to serve as the primary basis for conclusions. Our primary objectives were centered on item-level analyses, which more accurately reflect psychometric quality and facilitate the detection of flawed items.

At the item level, only 0.6% required corrections (Table 2), with the most common causes being ‘multiple-answers’ (~ 47%) and ‘wrong-answer’ (~ 41%) causes. The ‘item-removal’ cause likely stemmed from missing or incomplete exam questions and the absence of correct answers, leading to a lack of item *p*- and r_pb_-values, all of which should not occur in exams. Therefore, careful consideration of exam analysis is important to ensure that each question is complete, clearly conveys the intended message, and includes a definitive correct answer, so that students’ marks truly represent their capabilities. Consistent with prior work, expert review is essential for screening out invalid items, including questions that lack clarity, encourage guessing, or fail to test relevant knowledge, so that only sound and meaningful items are retained in the exam [16, 17]. This helps to avoid giving higher or lower marks than students deserve, which undermines the learning process.

Notably, all flawed items arose from technical or item-construction issues, such as miskeyed correct answers, multiple correct answers, or incomplete stems, and were not related to changes in recommended medical practice. Previous studies have also identified technical flaws such as miskeyed answers, unclear or ambiguously worded stems, and inadequate student preparation, rather than evolving clinical knowledge [18]. This indicates that the irregular psychometric patterns detected in our dataset were not attributable to evolving clinical guidelines but rather to errors in exam item design and verification.

When examining the characteristics of corrected and uncorrected items in detail, only items from the most recent academic year (2022) were reviewed due to the large amount of data (Table 3). Among all items analyzed, only 0.4% required correction. The mean item *p*- and r_pb_-values of corrected items were significantly lower than those of uncorrected items, indicating that corrected items were more difficult and less discriminative. Notably, negative item r_pb_-values were still present among the uncorrected items, similar to those observed among the corrected items, although the absolute values were much lower. This highlights the importance of not relying solely on the r_pb_-value threshold, as items with slightly negative discrimination indices may still function adequately within an exam if supported by other quality indicators. Therefore, a holistic item review approach, integrating both quantitative indicators and expert judgment, remains essential to ensure fairness, validity, and accuracy in MCQ-based assessments, with practical implications for routine exam review [17]. While this principle is widely acknowledged, our study contributes by empirically quantifying how often flawed items are missed when relying solely on psychometric thresholds, thereby reinforcing the importance of integrating expert judgment with statistical indicators in routine exam evaluation.

Item correction status exhibited negative correlations with both item *p*- and r_pb_-values (Table 4), confirming the established understanding that flawed items tend to be more difficult and less discriminative, which is consistent with a prior study [19]. The item *p*-value also exhibited a positive correlation with the item r_pb_-value. However, exam correction status did not show a significant correlation with either the mean exam *p*-value or the mean exam r_pb_-value. This may be explained by the fact that exams typically consist of a mix of items with varying difficulty and discrimination indices, so the presence of a small number of flawed items does not substantially influence the overall mean *p*- or r_pb_-values of the entire exam. Nevertheless, the mean exam *p*-value still showed a positive correlation with the mean exam r_pb_-value (Table 4). This consistent positive relationship between *p*- and r_pb_-values, observed at both the item and exam levels, may reflect that exams in which students were generally able to answer correctly (higher *p*-value) still contained items capable of differentiating student performance (higher r_pb_-value). In other words, exams that were not overly difficult allowed students to demonstrate their abilities more clearly, thereby maintaining discrimination between higher- and lower-performing examinees. It should be noted that the *p*-value is a component of the r_pb_-value formula; therefore, these two indices are not independent. As such, the correlation between them may be structurally inflated. While the relationship was statistically significant, the correlation coefficients were weak, suggesting that the association is of limited practical significance and should be interpreted with caution.

Considering all corrected items, most of them had a *p*-value below 0.3 (~ 85%) (Fig. 2A) and an r_pb_-value below zero (~ 50%) (Fig. 2B); however, 1 corrected item (~ 1%) (Fig. 2A) fell into the easy item *p*-value range (*p*-value > 0.7), and 4 corrected items (~ 5%) (Fig. 2B) fell into the good r_pb_-value range (r_pb_-value ≥ 0.25), indicating that flaws may still occur even in items with acceptable difficulty and discrimination indices. When considering both item *p*- and r_pb_-value ranges together, most corrected items fell within *p*-value < 0.3 and r_pb_-value < 0 (~ 47%) (Fig. 2C), and *p*-value < 0.3 and r_pb_-value 0–<0.25 (~ 35%), indicating that *p*-value was the most important factor for detecting flawed items, while the r_pb_-value contributed less than the *p*-value. This suggests that a low item *p*-value is a stronger signal of item flaws, likely because poorly constructed items tend to confuse most students regardless of ability. This aligns with evidence that distractor plausibility is influenced by item difficulty. Very easy items often yield non-functioning distractors [20, 21], while excessively difficult items increase the chance of multiple plausible distractors, lowering discrimination [22]. Thus, thresholds based solely on the *p*-value may fail to account for the interaction between difficulty and distractor functioning.

When corrected items were categorized by *p*- and r_pb_-value thresholds, detection of flawed items was ~ 80% for the *p*-value threshold (*p*-value < 0.25) (Table 5), ~ 50% for the r_pb_-value threshold (r_pb_-value < 0), and ~ 85% when applying either or both thresholds, indicating that the *p*-value threshold detected more flawed items than the r_pb_-value threshold. However, even using both thresholds still missed about 15% of flawed items.

For item corrections categorized by causes and thresholds, the most common *p*- and r_pb_-value ranges for each cause were found at *p*-value < 0.25 and r_pb_-value < 0 for the ‘wrong-answer’ cause (Fig. 3), and at *p*-value < 0.25 and r_pb_-value ≥ 0 for the ‘multiple-answers’ and ‘all-choices’ causes, indicating that corrections for the ‘wrong-answer’ cause tended to result from items that were more difficult and less discriminative, while corrections for the other causes were largely due to overly difficult items.

The higher item *p*-value of the actual correct answer, compared to the initially designated answer for the ‘wrong-answer’ cause (Fig. 4A) or the additional correct answer for the ‘multiple-answers’ cause (Fig. 4C), suggests that items in which the initially designated correct answer has a *p*-value lower than other options should be carefully reconsidered for potential key errors or the presence of multiple correct answers. Furthermore, this pattern may reflect unclear wording, excessive difficulty, or student misunderstanding, and prioritizing the review of such items could enhance exam validity and fairness.

The mean item r_pb_-value of the initially designated correct answer was negative and significantly lower than that of the actual correct answer for the ‘wrong-answer’ cause (Fig. 4B), whereas it was comparable to the additional correct answer(s) for the ‘multiple-answer’ cause (Fig. 4D). These findings suggest that the negative r_pb_-value of the initially designated correct answer warrants careful consideration. This is consistent with prior evidence that item-writing flaws related to the stem and choices negatively affect psychometric quality, leading to higher difficulty and lower discrimination [19].

Moreover, even when the r_pb_-values of the initially designated correct answer do not reveal apparent abnormalities and demonstrate good discrimination indices, there may still be problems, such as having more than 1 correct answer, especially when its *p*-value is comparable to or lower than those of other choices. This might stem from distractors that are not carefully evaluated, which could inadvertently become additional correct answers. Since almost all MCQ items consisted of 1 correct option and 4 distractors, with a few items containing 3 distractors, this could limit further analysis of distractor number variation; however, it highlights the importance of ensuring that all distractors are plausible and that non-functional distractors are minimized.

Large-scale analyses of distractor efficiency have demonstrated that item difficulty increases as functioning distractors are added but reaches a plateau beyond 3 distractors [22]. In contrast, item discrimination follows a curvilinear pattern, attaining its peak with approximately 4 functioning distractors and subsequently declining with additional distractors [22]. These findings emphasize that the plausibility of distractors is central to item quality, underscoring the need to prioritize the construction of high-quality distractors over their quantity in exam design [22]. The authors suggest that instructors should meticulously review distractors to ensure they cannot be interpreted as additional correct options, especially in challenging questions, to avoid items that could unintentionally function as having more than one correct answer if not carefully reviewed.

Collectively, threshold-based indicators are useful, but we acknowledge that static thresholds alone may miss some flawed questions, resulting in under-detection of about 15% of flawed items. This supports prior recommendations that item analysis should be supplemented with expert review, since relying only on statistical cutoffs risks overlooking flawed items [16, 17]. To strengthen detection, complementary strategies may be adopted, including response-pattern analysis to flag items where distractors outperform the keyed answer, peer review by other instructors, increased faculty awareness during question construction, integration of automated tools to support large-scale detection, and pretesting of new items prior to formal examinations [16, 17]. In addition, structured faculty training and periodic audits of item banks can help prevent recurrence of flawed items and promote continuous quality improvement [23]. This combined quantitative and qualitative approach provides a more comprehensive and practical framework for improving the overall quality and validity of MCQ exams.

In response, the Education Department has established guidelines for appropriate exams and set up a central committee to develop and review exam questions. This ensures that all exam questions adhere to the guidelines for effective and complete exam questions, including alignment with the course learning outcomes (CLOs), compliance with the Medical Competency Assessment Criteria for the National License, appropriate difficulty levels, a good discrimination index, and the provision of only 1 correct answer. Furthermore, incorporating future guidelines to flag items whose initially designated correct answer has a *p*-value comparable to or lower than that of other options may further enhance the accuracy of exam evaluation.

## Conclusions

This proof-of-concept study, drawing on a large, real-world dataset of preclinical MCQ exams, provides empirical evidence on how flawed items can be characterized and detected. Although only 0.6% of items required correction, the most frequent flaws were multiple correct answers and incorrect key designation. Low item difficulty was the most consistent signal of flawed items, while discrimination indices alone were less reliable. Thresholds based on *p*- and r_pb_-values identified most problematic items but still missed a notable proportion, highlighting the limitations of relying solely on static thresholds. These findings underscore the value of combining psychometric thresholds with expert review and structured quality assurance to improve exam validity. Future efforts should integrate response-pattern analysis, prospective item review, and faculty training to balance detection accuracy with feasibility in routine exam practice.

### Limitations

In medical year 1, only a few exam analyses were conducted for the 4 basic medical sciences courses that were transferred from medical year 2 of the 2017 curriculum to medical year 1 in the academic year 2021. This change allowed for the analysis of medical year 1 exams to be conducted only after these adjustments. Furthermore, following the outbreak of COVID-19, most exams were conducted separately by each course in small segments, resulting in most exams not being submitted to the Education Department for analysis.

## Data Availability

The datasets used and/or analysed during the current study are available from the corresponding author on reasonable request.
